# Defining the end of puberty in boys: INSL3 and the acute determinants of adult Leydig-cell functional capacity

**DOI:** 10.3389/fendo.2025.1574760

**Published:** 2025-05-27

**Authors:** Bilal Tulumcu, Richard Ivell, Waleed Alhujaili, Ravinder Anand-Ivell

**Affiliations:** School of Biosciences, University of Nottingham, Sutton Bonington, United Kingdom

**Keywords:** ALSPAC, INSL3, Leydig cell, hypogonadism, puberty

## Abstract

**Introduction:**

Testicular Leydig cells are responsible for producing almost all the testosterone required by men throughout the lifespan, with reduced testosterone (hypogonadism) correlating with age-linked morbidity and mortality. Leydig cells derive from stem cells within the testes after birth. These undergo proliferation and differentiation during puberty to achieve their final adult status in young adulthood, after which there appears to be no further cell division and only very limited attrition into old age. Leydig-cell functional capacity reflects the total number and differentiation status of the Leydig-cell population within an individual and can be assessed by measuring in blood the constitutive Leydig-cell hormone insulin-like peptide 3 (INSL3). In adult men, this varies by more than 10-fold between individuals and correlates with later morbidity. Such INSL3 variance appears to have its origin already in young men, though what determines this is largely unknown.

**Methods:**

Here, we have used the ALSPAC (Avon Longitudinal Study of Parents and Children) cohort of boys and young men to estimate when the adult-type Leydig-cell population becomes established, that is, when puberty ends, and the contemporary anthropometric and lifestyle parameters that influence this.

**Results and discussion:**

At 17 years, mean INSL3 is not yet maximal, with high variance due to both longitudinal (timing of pubertal trajectory) and cross-sectional influences, whereas at 24 years, circulating INSL3 concentration has stabilized to its final adult status, even showing a small decreasing trend with age. Maximal INSL3 (i.e., peak puberty) was calculated to be at approximately 22 years in this cohort. Both contemporary body mass index and smoking status, though not inflammatory parameters, were contributory factors to INSL3 concentration. However, the major source of INSL3 variance in young men was shown to be already established at 17 years, with causative influences evidently occurring prior to this age, and showing that early life parameters are important for determining later adult health in men.

## Introduction

Puberty in boys represents the delayed postnatal establishment of the HPG axis, with the production of circulating testosterone and initiation of spermatogenesis, beginning earliest at approximately 9–10 years of age. Anatomically, pubertal development in boys is monitored by Tanner staging of pubic hair growth and testis size, as well as by closure of the long bone hypophyses. Because these anatomical indices may be temporally variable and imprecise, other indices to assess pubertal development have been characterized, such as age at peak height velocity (APHV), which tries to capture the main pubertal growth spurt, or the timing of voice break, to offer parameters with more precision (1). However, it is still unclear when the phase of pubertal development ends and adulthood begins and how this may vary.

Largely based on animal experiments, pubertal development begins in the interstitial compartment of the testes with the proliferation and differentiation of Leydig stem cells, which are fusiform mesenchymal cells located around the tunica of the seminiferous tubules. Concomitant with the proliferation and differentiation of germ cells within the seminiferous tubules, these Leydig stem cells give rise to a population first of progenitor cells, then immature Leydig cells, and finally adult-type mature Leydig cells (2, 3). In the adult rat, these comprise about 50 percent of all interstitial cells alongside macrophages and dendritic cells, lymphoid cells, vascular endothelial cells, smooth muscle cells, and mesenchymal cells (4). At the end of puberty in the paired human testes, there is a mature population of approximately 200–250 million Leydig cells (5, 6), which are mostly terminally differentiated, have stopped dividing, and are responsible for the production of approximately 95% of all testosterone required to support male physiology throughout the remainder of the lifespan.

The production of testosterone by the Leydig cells is driven by pulses of LH from the pituitary, which during pubertal development serve both to induce Leydig-cell proliferation as well as differentiation, but at the end of puberty only act acutely to stimulate the enzymes and factors responsible for testosterone synthesis within the now stable population of Leydig cells. In humans, puberty is characterized by the frequency and amplitude of these LH pulses and the consequent testosterone surges (“storm”) until full puberty is attained (7). These then settle down to a lower pulsatile steady state, maintained by homeostatic feedback of the steroid to the brain and pituitary. From early adulthood onwards, circulating testosterone levels are homeostatically maintained into older age by increasing LH production, with minimal reduction in average testosterone levels, although there is still some diurnal rhythmicity with increasing loss of LH-testosterone synchronicity (8).

Mature Leydig cells also secrete another hormone, INSL3. This peptide hormone is produced and secreted constitutively with little or no diurnal or other short-term variation (9). The circulating concentration of INSL3 is determined uniquely by the numbers and mature differentiation status of the Leydig cells of the testes and effectively measures their total functional capacity (10). Except for a small postnatal increase during the so-called “mini-puberty” at 1–6 months of age (11), INSL3 is undetectable in blood before puberty onset and increases gradually during puberty, acting as a biochemical Tanner staging to monitor and quantify pubertal progression (10, 12). Although there is very little within-individual variance in circulating INSL3 concentration once adulthood is reached, there is more than a 10-fold variation in levels between individuals in community-dwelling men (13–15). In middle-aged and older men, individual INSL3 values correlate with and predict morbidity (16), in part because they reflect the capacity of the testes to produce androgens, with low INSL3 (<0.4 ng/ml) being a good index for clinical hypogonadism (17). Because of the consistency of INSL3 concentration within any one individual over long periods of several years, it seems likely, therefore, that whatever determines the final level of INSL3 in the circulation in young men at the end of puberty may be predictive of their later health and morbidity. This level most likely reflects the result of those events in childhood that determine the relative rates of Leydig precursor cell proliferation and differentiation, as well as the systems that regulate these, about which we currently have very little understanding. Moreover, in the 18-year-old young men from a Swedish cohort, we could find no correlation at all between circulating INSL3 concentration and contemporary semen parameters (14). Hence it would appear that FSH concentration and, by extrapolation, those factors influencing the hypothalamo-pituitary end of the HPG axis are unlikely to be instrumental in acutely determining individual Leydig-cell functional capacity, which includes their ability to produce testosterone.

The present study takes advantage of the ALSPAC (Avon Longitudinal Study of Parents and Children) longitudinal cohort drawn from the Bristol region of the UK (18, 19). Blood samples were collected and analysed for INSL3 from boys/young men within this cohort at approximate ages of 17 and 24 years. Our main objectives were, firstly, to determine whether INSL3 varied across this population of young men in a similar manner as has been determined in other cohorts and how this might change towards the end of puberty between 17 and 24 years in the important final establishment phase. Secondly, the longitudinal nature of this cohort allows us to examine a wide range of possible factors during childhood and adolescence that may contribute to the final Leydig-cell functional capacity as represented by INSL3 concentration in young adulthood.

## Methods and materials

The ALSPAC is a prospective, population-based birth cohort study conducted in the United Kingdom. Established to investigate the determinants of health and development across the lifespan, ALSPAC enrolled pregnant women residing in the former county of Avon with expected delivery dates between April 1991 and December 1992 (18, 19). The initial cohort comprised 14,541 pregnancies. This was supplemented by further children recruited at around age 7, so that the total sample size for analysis of data collected post 7 years reflected a total of 15,447 pregnancies. Of these, 14,901 children were alive at 1 year of age. Comprehensive data have been collected from participants through questionnaires, clinical assessments, biological samples, and linkage to medical records (for more details see, www.bristol.ac.uk/alspac/researchers/our-data/).

In the present study, we utilized data from 1,781 young men within the ALSPAC cohort. Blood analyses, anthropometric measurements, clinical assessments, and questionnaire data were collected when these participants were approximately 17 and 24 years old (20). For the 24-year-old subjects, data were collected and managed using REDCap electronic data capture tools hosted at the University of Bristol (21). REDCap (Research Electronic Data Capture) is a secure, web-based software platform designed to support data capture for research studies. Subjects had been excluded who had a defined congenital illness (e.g., trisomy) or who had at some stage in their lives had one or both testes removed.

### Measures

During the 2-hour clinical assessments at ages 17 and 24, participants underwent various measurements and tests. Height was measured by a Harpenden stadiometer. Weight was assessed with a Tanita Body Fat Analyser (Model TBF 401A). Lunar dual-energy X-ray absorptiometry (DXA) scans were performed to conduct whole-body assessments, providing detailed measurements of bone content, lean mass, and fat mass. Additionally, questionnaires were administered during these clinical visits to collect data, particularly regarding smoking consumption. Age at peak height velocity (APHV) was calculated according to Golding et al. (1).

### Blood samples

Peripheral venous blood samples were collected during these clinical visits; serum or plasma was prepared, aliquoted, and stored frozen at −80°C until analyses. At 17 years, these were analysed for C-reactive protein (CRP), cotinine, and INSL3. At 24 years, these were analysed for CRP, the inflammatory markers interferon-γ (IFNγ), interleukins-6 (IL-6) and -8 (IL-8), tumour necrosis factor-α (TNFα), and white blood cell count (WBC), as well as INSL3. CRP was measured by automated particle-enhanced immunoturbidimetric assay (Roche Diagnostics, Burgess Hill, UK). Cotinine was measured using the Cozart Cotinine Enzyme Immunoassay (Concateno (now Alere Toxicology), Abingdon, UK) serum kit (M155B1). IFNγ, IL-6, IL-8, and TNF-α were assayed in heparin plasma samples using Olink Proteomics’ Proximity Extension Assay (PEA) technology (Olink Proteomics, Uppsala, Sweden), with data represented as normalized protein expression values, which are Olink Proteomics’ arbitrary units on a log_2_ scale. INSL3 was measured using a well-validated time-resolved fluorescent immunoassay as previously used for other large cohort studies (13–15). The limit of detection for this assay was 20 pg/ml, inter- and intra-plate coefficients of variation were <9% and <3%, respectively, and this assay shows no cross-reactivity with any other insulin- or relaxin-like molecules (9).

### Smoking habits

Smoking was assessed by blood cotinine measurement at age 17 years and by self-reported questionnaires at ages 17 and 24 years. Questions included the following: “at what age did you smoke your first cigarette?” “have you smoked in the last 30 days?” “what was your age when you last smoked?” “do you smoke every day?” “number of cigarettes smoked per day” “do you smoke every week?” “number of cigarettes smoked per week,” “do you vape every day?,” “how long have you been vaping?,” and “what is the frequency of vaping?” “Regular smokers” were defined as those boys who agreed to smoking at least daily or weekly; “non-smokers” were defined as those who responded as never smoking. This categorization therefore does not include occasional or casual smokers, smoking less than weekly. These questionnaires were only completed by a smaller number of subjects, limiting the statistical power of the resulting analyses.

### Statistical analysis

All statistical analyses were conducted using R software (version 2024.05.0), GraphPad Prism (Version 10.3.1), or SPSS (Version 29.02.0). Descriptive statistics were calculated for participants at ages 17 and 24 years. Variables indicating significant skewness were log-transformed to approximate normality prior to analysis. Simple bivariate correlation analyses were performed to assess relationships between INSL3 concentration at ages 17 and 24 years and available scalar parameters. Multiple regression analysis was carried out for available non-collinear parameters, testing stepwise with *p* < 0.10 for inclusion and *p* < 0.05 for exclusion. Independent t-tests were utilized to compare mean INSL3 levels between groups based on questionnaire responses, using Welch’s correction for unequal variance. All statistical tests were two-tailed, and a *p*-value less than 0.05 was considered to imply statistical significance.

### Ethical considerations

The study was approved by the ALSPAC Ethics and Law Committee and the Local Research Ethics Committees. Consent for biological samples was obtained from all participants, in accordance with the Human Tissue Act 2004. Informed consent for the use of data collected via questionnaires and clinics was obtained from participants following the recommendations of the ALSPAC Ethics and Law Committee at the time. All data were fully anonymized. Detailed descriptions of the cohort and study design have been previously published and are accessible on the ALSPAC website (www.bristol.ac.uk/alspac/researchers/our-data/), which also provides comprehensive details of all available data through a fully searchable data dictionary and variable search tool.

## Results

### Circulating INSL3 at ages 24 and 17 years from the ALSPAC cohort

**Figure 1:**
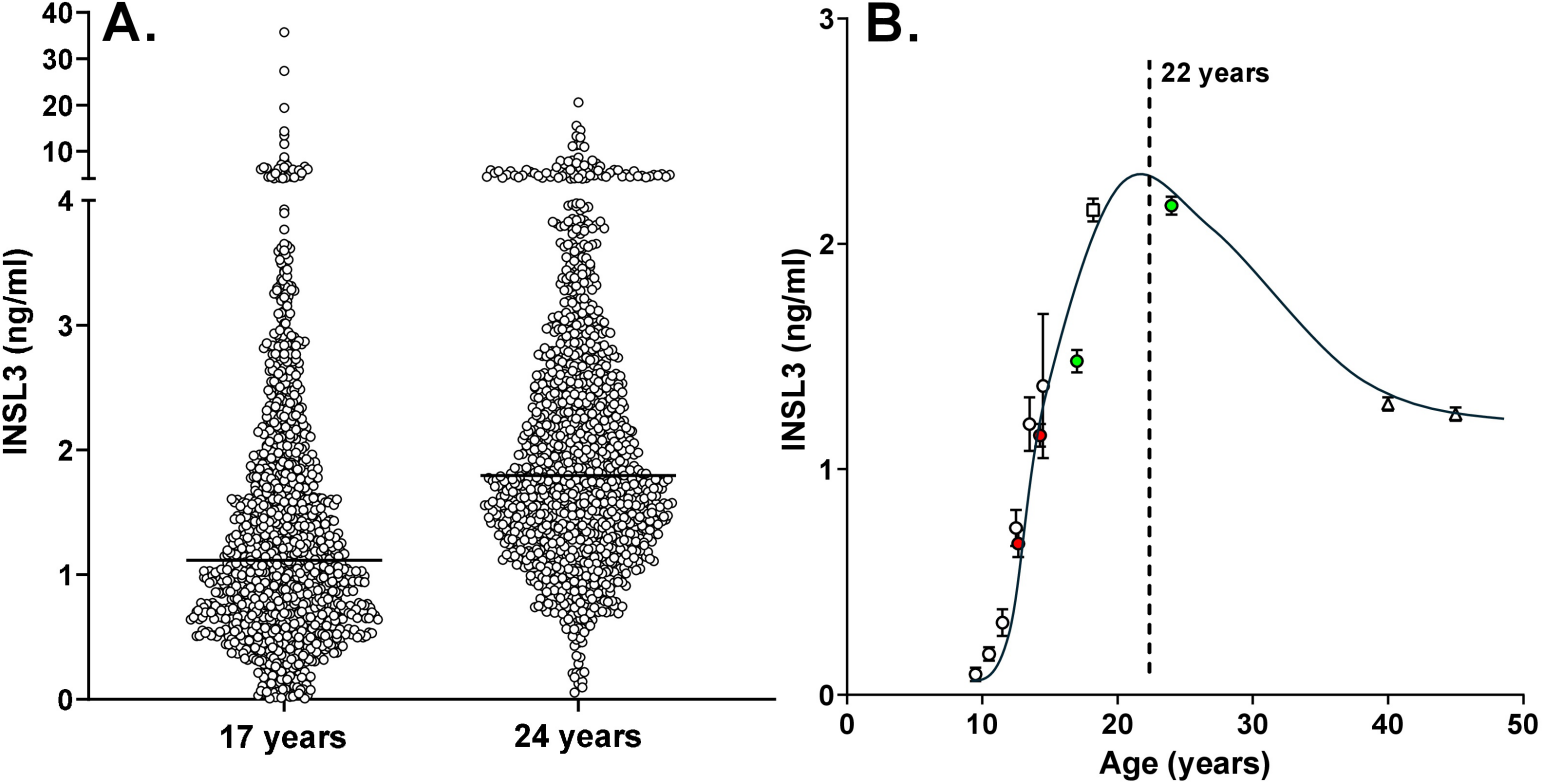
**(A)** Scatterplots of individual circulating INSL3 concentration in boys from the ALSPAC cohort at 17 years (*n* = 1,250) and 24 years (*n* = 1,177) of age. The horizontal line indicates the median for each age group. **(B)** Collated means ± SEM for circulating INSL3 in largely Caucasian populations of boys and men at specific ages. Data from the present study are represented by the green data points. Also included are data for control groups of healthy boys at Tanner stages 2 and 4 (red data points) (22), and our own studies using the same TRFIA assay as in the present study on boys in puberty (12) (white circles), at 18.2 years of age (14) (white square), and middle-aged community-dwelling men (<50 years) from the European Male Aging Study (EMAS) (15) (white triangles; because of the large number of subjects included, standard errors are smaller than the symbols).

**Table 1** shows the available descriptive statistics for the 24- and 17-year-old male members of the ALSPAC cohort. Mean INSL3 at 24 years is significantly greater than at 17 years (**Table 1**, **Figure 1**) and comparable to the mean INSL3 level estimated for a Swedish cohort of military conscripts of mean age 18.2 years (14). The variance at both ages is also similarly high, with a range of approximately 100-fold, agreeing with the notion that circulating INSL3 concentration and hence Leydig-cell functional capacity may not yet be stabilized to a final adult state.

**Table 1:** Descriptive statistics of the 17-year and 24-year boys from the ALSPAC cohort.

	17 years			24 years		
	Mean ± SD	Median (25%, 75%)	*N*	Mean ± SD	Median (25%,75%)	*N*
Age (months)	213.1 ± 4.5	213 (211, 215)	1545	294.5 ± 9.4	294 (288, 301)	1,238
Height (cm)	178.9 ± 6.8	178.9 (174.4, 183.3)	1513	180.0 ± 6.7	179.9 (175.3, 184.3)	1,231
Weight (kg)	72.0 ± 12.8	70.3 (63.2, 78.3)	1514	80.3 ± 14.8	78.1 (70.0, 87.7)	1,230
BMI	22.5 ± 3.7	21.7 (20.7, 24.1)	1512	24.8 ± 4.3	24.1 (21.9, 26.8)	1,230
Waist circ (cm)	–	–	–	86.0 ± 11.4	83.5 (78.2, 90.7)	1,228
INSL3 (ng/ml)	1.48 ± 1.79	1.12 (0.68, 1.82)	1250	2.16 ± 1.55	1.80 (1.32, 2.57)	1,177

**Figure 2:**
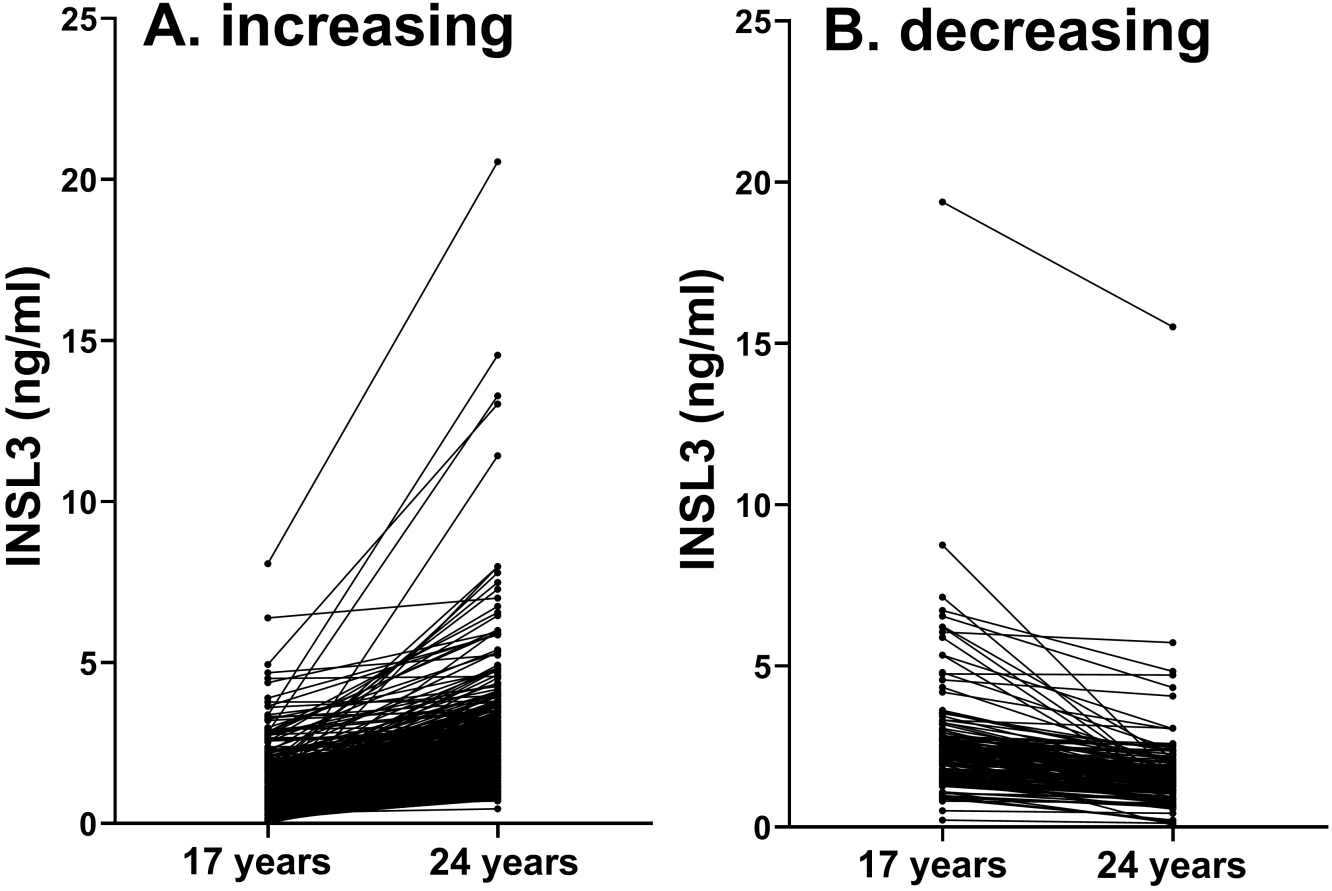
For those 645 individuals presenting at both 17 and 24 years, **(A)** represents those with increasing (*n* = 491) and **(B)** those with decreasing (*n* = 154) circulating INSL3 concentration between the 2 time points.

Although nominally 24 and 17 years of age, the recruited ALSPAC boys and young men indicated a range of 40 and 30 months, respectively, at the time of blood collection and parameter assessment. Regression analysis of the 24-year-old men with actual age indicated a downward trend at this time point ([INSL3] = −0.0192*[age in months] + 7.824; *p* < 0.0001; *n* = 1176), showing that Leydig-cell functional capacity was already showing the age-dependent decline from the post-pubertal maximum. This is more evident at older ages (13, 15) and thus implies that the average post-pubertal peak must be prior to 24 years. In contrast, there is no such correlation with age for the 17-year time point, emphasizing that at this age not only is the post-pubertal maximum not yet attained, but also factors influencing final adult INSL3 levels are still active. Blood samples from approximately 1,200 boys each at 17 years and 24 years were available, but only 645 boys presented at both time points. Of these, 491 indicated an increase in INSL3 concentration between 17 and 24 years, while 155 showed a decrease (**Figure 2**). From the ratio of these two categories, it can be estimated that peak INSL3 is reached at approximately 22 years of age, allowing a smoothed curve of mean INSL3 to be drawn as in **Figure 1B** (ALSPAC data represented by the green data points). This figure also includes data from another study by Taneli et al. (22) or control groups of healthy boys at Tanner stages 2 and 4 (red data points), and our own studies on boys in puberty (12), at 18.2 years of age (14), and middle-aged community-dwelling men from the European Male Aging Study (EMAS) (15) (**Figure 1B**, white data points).

### Sources of INSL3 variance at 24 and 17 years

**Figure 3:**
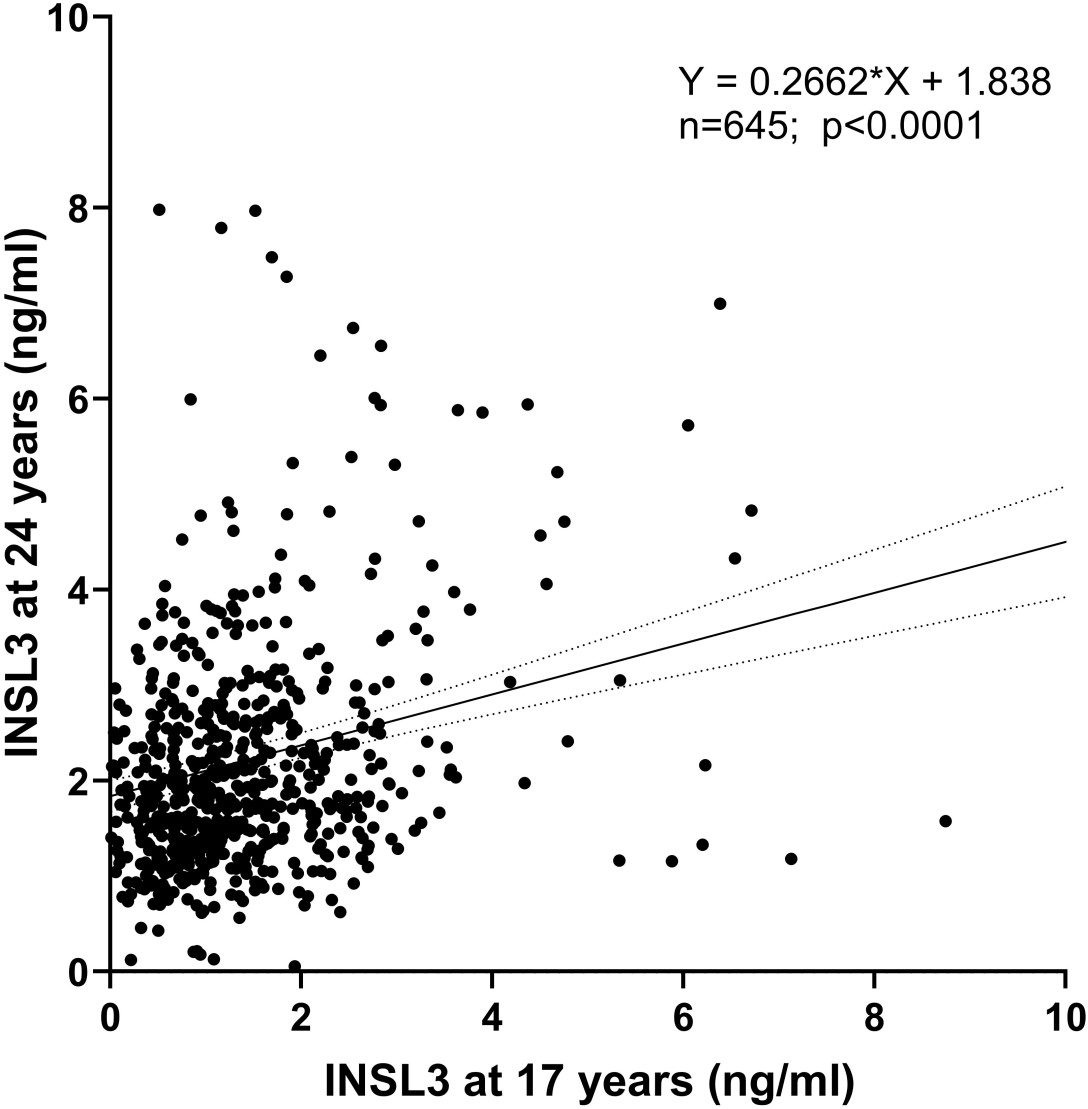
Regression of circulating INSL3 concentration measured at 17 years (x-axis) and 24 years (y-axis) for those individuals presenting at both times.

First, for the 645 boys who presented at both 17 and 24 years, there is a significant correlation (*p* < 0.0001) in their INSL3 values (**Figure 3**). This shows that despite other factors likely influencing the final 24-year-old Leydig-cell functional capacity, the status at 17 years of age, when puberty is still not complete, is a key factor in later young adult status. Bivariate regression analysis of the log-transformed INSL3 concentration at 24 years against contemporary biochemical and anthropometric parameters (**Table 2**) showed effects of real age in months, lean body mass, though not bone or fat mass, determined by DEXA, as well as of weight, body mass index (BMI), and waist circumference. There was no relationship to height alone, suggesting that growth per se is not a relevant factor. There was no relationship to any of the inflammatory markers, including CRP, though these correlated well amongst themselves (**Supplementary Table S1**) as also with BMI (**Supplementary Table S2**). These relationships were not affected by correction for real age in months (not shown). In contrast, for the approximately 1,200 boys assessed at 17 years, of such parameters, only BMI indicated a negative relationship, and then with a greater variance (and hence *p*-value) than at 24 years (**Table 2**).

**Table 2:** Bivariate correlation analysis of INSL3 at 17 and 24 years versus available scalar parameters.

INSL3 at 24 years				INSL3 at 17 years		
	Pearson correlation	Significance	*N*	Pearson correlation	Significance	*N*
INSL3 at 17	0.243	< 0.001	645			
CRP	−0.020	0.513	1,064	-0.009	0.741	1,242
IFNγ	0.011	0.719	1,031	–	–	–
IL-6	−0.023	0.451	1,031	–	–	–
IL-8	0.046	0.144	1,031	–	–	–
TNFα	0.044	0.163	1,031	–	–	–
WBC	−0.057	0.054	1,159	–	–	–
Age (mo.)	−0.137	< 0.001	1,176	0.038	0.184	1,250
Bone mass	−0.041	0.169	1,143	−0.032	0.266	1,212
Fat mass	−0.011	0.714	1,143	−0.018	0.523	1,212
Lean mass	−0.113	< 0.001	1,143	−0.041	0.157	1,212
BMD	–	–	–	−0.014	0.624	1,212
Height	0.031	0.296	1,169	0.048	0.091	1,227
weight	−0.081	0.006	1,168	−0.032	0.264	1,227
BMI	−0.101	< 0.001	1,168	−0.059	0.038	1,226
Waist circ.	−0.071	0.016	1,166	–	–	–
APHV^1^	−0.018	0.578	1,002	−0.067	0.024	1,150
Smoking^2^	−0.152	0.002	398	–	–	–
Smoking^3^	−0.033	0.767	82	−0.157	0.043	167
Cotinine	–	–	–	0.039	0.279	770

CRP, c-reactive protein; WBC, white blood cell count; BMD, bone mineral density; BMI, body mass index; APHV, age at peak height velocity. ^1^The correlation with INSL3 at 17 years becomes insignificant when corrected for actual age in months. ^2^Smoking assessed as “age when last smoked”. ^3^Smoking assessed as “number of cigarettes smoked per week.” Red text indicates significant correlations.

### Influence of smoking on INSL3 concentration

The bivariate correlation analysis indicated a correlation at 24 years between INSL3 concentration and the scalar parameter from the questionnaire “age when last smoked” (**Table 2**). Similarly, at 17 years of age there was a negative correlation between INSL3 concentration and the scalar parameter “number of cigarettes smoked per week,” though the *n*-value is relatively small. For the more objective parameter, cotinine concentration measured at 17 years, there was no such correlation.

The questionnaires also generated various categorical parameters, which could be tested by simple comparative statistics (e.g., Welch’s t-test for unequal variance) (**Table 3**). Although at 17 years these questionnaire parameters behaved as expected in relation to blood cotinine concentration (**Supplementary Table S3**), they showed no relationship to INSL3 concentration, though did differ at 24 years (**Table 3**). We also checked whether there was an age difference between “regular smokers” and “never smokers,” since this could account for some of the difference in INSL3 concentration at 24 years (see above). There was indeed a small difference in age (mean 1.8 months) between these smoking categories; however, when calculated, this could only account for maximally 4% of the difference in INSL3. When a cotinine value of 3.0 ng/ml, which represents the U.S. and U.K. cutoff for smokers versus non-smokers (23), is applied and a simple t-test used to compare those >3.0 ng/ml (30.8% of the available total) versus those with cotinine <3.0 ng/ml, there is no significant difference in mean INSL3 concentration at 17 years (**Table 3**). Similar questionnaire parameters were also evaluated at 24 years for vaping, though numbers were small. No effect of vaping either alone or together with cigarette smoking was evident at this age on the circulating INSL3 concentration (not shown).

**Table 3:** Relationship between smoking status and peripheral INSL3 concentration.

	**INSL3 at 17 years (ng/ml)**			**INSL3 at 24 years (ng/ml)**		
	Mean ± SD	*n*	Significance	Mean ± SD	*n*	Significance
Cotinine _=_ 3.0 ng/ml	1.52 ± 2.55	235	ns	na		
Cotinine < 3.0 ng/ml	1.37 ± 1.70	535		na		
Regular smokers	1.45 ± 1.32	169	ns	1.95 ± 1.37	225	*P* < 0.001
Never smoked	1.30 ± 0.98	105		2.32 ± 1.70	681	

Even when separated into quartiles, cotinine at age 17 still does not indicate an effect of smoking on INSL3 concentration (not shown). “regular smokers” are those that indicated in questionnaires that they smoked at least weekly or daily; “never smoked” were those that amongst the remainder indicated that they had never smoked a single cigarette. Occasional smokers (less than weekly or only occasionally) were not included in the analysis. ns, not significant; na, not applicable, no data.

### Influence of pubertal trajectory on INSL3 concentration

**Figure 4:**
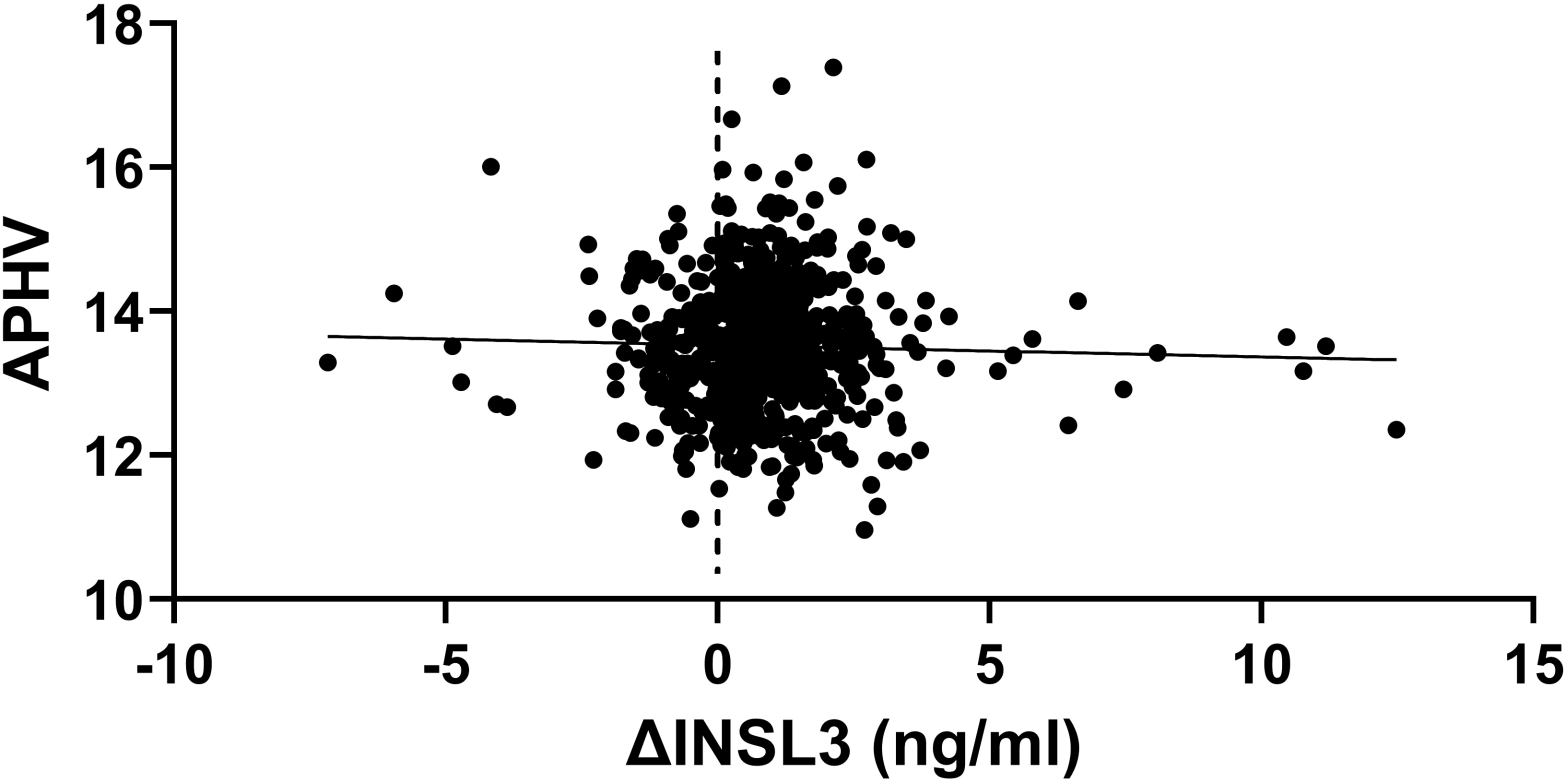
Scatterplot of the difference in INSL3 between 17 and 24 years (δINSL3) against the age at peak height velocity (APHV) as a measure of the pubertal trajectory (*n* = 645).

It is possible that much of the variance in INSL3 values at the end of puberty might be caused by differences in timing of the pubertal trajectory, with some boys entering puberty earlier or later than others, and thus potentially shifting the mean trajectory curve (**Figure 1**) to the left or right. To assess this aspect, we carried out simple regression analysis (**Figure 4**) of the change in INSL3 (ΔINSL3) concentration for the 645 boys presenting at both 17 and 24 years against the estimated age at peak height velocity (APHV) as an independent measure of pubertal timing (1). No relationship is evident. Nor is there any relationship between age at APHV and INSL3 at 24 years (**Table 2**). There appears to be a minor correlation for INSL3 at 17 years (**Table 2**), which however disappears upon correction for real age in months, suggesting that the final circulating INSL3 concentration at 24 years is independent of the earlier pubertal trajectory. We further compared APHV means for those boys whose INSL3 decreased between 17 and 24 years and those where this increased. There was no difference between the two subgroups (INSL3 decreasing between 17 and 24 years: APHV, 13.47 ± 0.79 years; INSL3 increasing between 17 and 24 years: APHV, 13.54 ± 0.95 years). It should also be noted that the change in INSL3 concentration is independent of any change in height between 17 and 24 years, which has increased only by approximately 1 cm in this time period (**Table 1**).

In fact, when a multiple regression analysis is undertaken of INSL3 at 24 years (**Table 4**), including the non-collinear contemporary parameters measured at 24 years (**Table 2**), as well as the INSL3 concentration at 17 years, only this last parameter is a contributor to variance, implying that Leydig-cell functional capacity is largely determined already at 17 years.

**Table 4:** Multiple regression analysis for INSL3 at age 24 years.

Included parameter	B ± SD	Significance	95% CI for B
Ln INSL3 at 17 years	0.184 ± 0.048	*P* < 0.001	(0.088, 0.279)

Parameters excluded: CRP, IFNγ, IL-6, IL-8, TNFα, WBC, age (months), lean, bone and fat mass, waist circumference, height, BMI, smoking. Abbreviations as in **Table 2**.

## Discussion

The ALSPAC cohort is one of the few longitudinal studies that allows a retrospective analysis of childhood and adolescent parameters with contemporary measurement of gonadal function in the important post-pubertal years. Moreover, with blood samples taken at both 17 and 24 years, it allows consideration of just when peak Leydig-cell functional capacity is attained at the end of puberty, here approximating to 22 years of age. The resulting INSL3 trajectory is essentially similar to that from a recent study of Danish boys and young men using a different assay (24). The latter study appears to indicate a lesser decline with subsequent age than shown in **Figure 1**, possibly due to the differing geography of the EMAS subjects and their reduced average INSL3. In the latter study, we evidenced marked regional variation in mean INSL3 amongst older men (15). These studies also all show a similar >10-fold range of INSL3 values between individuals during adulthood. Because of the large number of subjects in the present study, a few values exceed 3 standard deviations from the mean, thus appearing unusually high; no pathology appears associated with this.

Here, we wish to understand first the significance and contemporary influences on post-pubertal Leydig-cell functional capacity, as represented by circulating INSL3 at 17 and 24 years of age. Strong evidence suggests that blood INSL3 concentration at the end of puberty is potentially predictive of later morbidity (16). Most relationships of INSL3 with contemporary parameters are evident at 24 years of age (**Table 2**) and are very similar to those established for older-aged cohorts such as FAMAS (13) and EMAS (15), as well as for the younger Swedish cohort of military conscripts (14). Namely, circulating INSL3 correlates negatively with BMI or waist circumference and negatively with smoking as well as age. In addition, we show for the first time that INSL3 at 24 years is also correlated negatively with lean body mass, implying a possible relationship to bone and muscle development. *In vitro* and *in vivo* studies have shown that low INSL3 is associated with reduced bone mineral density (16, 25) and that INSL3 can act via its specific receptor RXFP2 directly on osteoblasts and osteoclasts to influence bone deposition (26), as well as on skeletal muscle stem cells (27). Although values for circulating testosterone are not available for the ALSPAC cohort at this age, because at older ages INSL3 also correlates with circulating testosterone (15), this relationship to bone and muscle growth may simply be due to altered levels of testosterone in these individuals. There is no relationship between INSL3 at 24 years and any of the inflammation-related markers, including CRP (**Table 2**). INSL3 at 24 years does almost correlate with white blood cell count (*p* = 0.056). Both CRP and the other cytokines measured, as well as WBC, do correlate with each other as well as with measures of overweight and waist circumference in this dataset (**Supplementary Table S1**), indicating that, in this cohort, although there is a clear relationship between general systemic inflammation, increased body fat, and obesity (28), inflammatory parameters, measured contemporaneously, do not appear to influence Leydig-cell functional capacity as represented by circulating INSL3 concentration. In contrast, it is known that systemic inflammation negatively correlates with testosterone or free testosterone levels both in animal models of inflammation (29) and in human studies (30, 31).

The single most important relationship appears to be the correlation between circulating INSL3 at 24 years and that at 17 years within the same individuals (**Figure 2**, **Table 4**), implying that the final Leydig-cell functional capacity is at least in part already established at 17 years, even though the high variance at this age suggests that this is still quite fluid. In fact, when intra-class correlation (ICC) analysis is applied to compare the INSL3 concentrations in the same young men at 17 and 24 years, the resulting consistency coefficient is only 0.367 (95% CI: 0.262–0.458), which is considered “poor.” This is markedly different from the ICC consistency coefficient within older men from the EMAS cohort measured twice for INSL3 using the same assay 4–5 years apart (0.871; 95% CI: 0.854–0.887; unpublished data), which is considered “good” to “excellent.” In contrast to what is seen at 24 years, circulating INSL3 at 17 years is only significantly and negatively associated with BMI and smoking frequency and no other measured contemporary parameter, including age, cotinine, or CRP (**Table 2**). As for the 24-year-old subjects, there is, however, a significant correlation between CRP at 17 years and measures of overweight (BMI, weight, DEXA parameters; **Supplementary Table S2**), implying that the contemporary inflammatory parameters themselves may not be instrumental in influencing Leydig-cell function.

Very few studies have tried to determine the causes for the variation in adult Leydig-cell numbers and differentiation status, which manifest as circulating INSL3 concentration and represent the Leydig-cell functional capacity to produce hormones such as androgens essential for maintaining health into old age. In rats, although maternal exposures (*in utero* and via lactation) have shown marked effects of endocrine-disrupting chemicals such as xenoestrogens or phthalates on the dynamics of Leydig-cell proliferation and differentiation during the course of puberty or equivalent processes in models of Leydig-cell ablation and recovery (32, 33), such effects are rarely evident in later adulthood (34). In some way Leydig-cell functional capacity is restored to control values. In young bulls, early prepubertal nutrition was shown to influence (accelerate) the pubertal trajectory of increasing INSL3 up to young adulthood, when effects of the intervention became no longer apparent (35). In other words, such experiments suggest that manipulations such as these may alter the timing and dynamics of puberty but do not appear to affect the final adult Leydig-cell functional capacity, though in none of these examples were subjects followed beyond young adulthood into older age.

Nevertheless, in the adult human population, INSL3 varies considerably between individuals, and this appears to have consequences for later health and morbidity (16). Taken together, the results of the present analysis suggest that other factors during childhood, adolescence, or earlier may be responsible for the final Leydig-cell functional capacity developed during puberty but manifest for most of the remaining male lifespan. Moreover, INSL3 measured at age 17 is still subject to high variance, unlike at 24 years, when INSL3 expression appears to have stabilized and better reflects contemporary adult parameters.

In terms of clinical application, circulating INSL3 in boys offers a simple biochemical reflection of pubertal development, specifically focusing on the Leydig-cell compartment and its capacity to generate androgens. However, despite its constitutive nature, as also indicated by Albrethsen et al. (24), its high between-individual variance limits more general diagnostic application at the present time, though a consideration of INSL3 thresholds could allow a differentiation in cases of delayed puberty (36), Klinefelter syndrome (37–39), or in older men for functional hypogonadism (17). However, it is proving valuable in individual cases to assess, for example, recovery following anabolic steroid misuse (40) or steroidal male contraception (41), where exogenous steroids necessarily suppress the hypothalamo-pituitary-gonadal axis and hence cause the Leydig-cell population temporarily to dedifferentiate, or following long-term gonadotropin stimulation (42). Further research on the factors influencing adolescent levels of INSL3 will no doubt offer other opportunities for its clinical application.

The main weakness of the present study is due to its retrospective nature, precluding the subsequent measure of other contemporary parameters, such as testosterone, or other time points during this important establishment phase of late puberty. Nevertheless, the results provide important new information concerning the Leydig-cell compartment in men, which, once established at the end of puberty, appears to remain more or less fixed into older age.

## Data Availability

The raw data supporting the conclusions of this article will be made available by the authors and the ALSPAC executive committee without undue reservation.
