# Supplementary material for: Defining the end of puberty in boys: INSL3 and the acute determinants of adult Leydig-cell functional capacity
Source: Front Endocrinol (Lausanne). 2025 May 27;16:1574760. doi: 10.3389/fendo.2025.1574760 (PMC12148858; doi:10.3389/fendo.2025.1574760)
Supplement: Supplementary file 1 [file Table1.docx]

**Suppl. Table 1**

**Bivariate correlation analysis for inflammation and obesity-related parameters for young men aged 24 years.**

|  |  | **CRP** | **IFNγ** | **IL6** | **IL8** | **TNFα** | **WBC** | **DEXA**  **bone** | **DEXA**  **fat** | **DEXA**  **lean** | **weight** | **WC** | **BMI** |
| --- | --- | --- | --- | --- | --- | --- | --- | --- | --- | --- | --- | --- | --- |
| **INSL3** | *correlation*  *p-value*  *n* | -0.020  0.513  1064 | 0.011  0.719  1031 | -0.023  0.451  1031 | 0.046  0.144  1031 | 0.044  0.163  1031 | -0.057  0.054  1159 | -0.041  0.169  1143 | -0.011  0.714  1143 | -0.113  <0.0011143 | -0.081  0.006  1168 | -0.071  0.016  1166 | -0.101  <0.001  1168 |
| **CRP** | *correlation*  *p-value*  *n* | 1 | 0.247  <0.001  938 | 0.526  <0.001  938 | 0.117  <0.001  938 | 0.215  <0.001  938 | 0.301  <0.001  1059 | 0.046  0.140  1051 | 0.351  <0.001  1051 | 0.104  <0.001  1051 | 0.293  <0.001  1072 | 0.346  <0.001  1071 | 0.349  <0.001  1072 |
| **IFNγ** | *correlation*  *p-value*  *n* |  | 1 | 0.377  <0.001  1031 | 0.192  <0.001  1031 | 0.383  <0.001  1031 | 0.123  <0.001  1015 | 0.087  0.006  1000 | 0.055  0.080  1000 | 0.075  0.018  1000 | 0.069  0.027  1024 | 0.048  0.127  1024 | 0.046  0.142  1024 |
| **IL6** | *correlation*  *p-value*  *n* |  |  | 1 | 0.285  <0.001  1031 | 0.358  <0.001  1031 | 0.428  <0.001  1015 | 0.063  0.048  1000 | 0.339  <0.001  1000 | 0.090  0.004  1000 | 0.285  <0.001  1024 | 0.333  <0.001  1024 | 0.298  <0.001  1024 |
| **IL8** | *correlation*  *p-value*  *n* |  |  |  | 1 | 0.388  <0.001  1031 | -0.016  0.606  1015 | 0.044  0.169  1000 | 0.068  0.032  1000 | 0.027  0.401  1000 | 0.055  0.078  1024 | 0.092  0.003  1024 | 0.056  0.072  1024 |
| **TNFα** | *correlation*  *p-value*  *n* |  |  |  |  | 1 | 0.098  0.002  1015 | 0.009  0.766  1000 | 0.148  <0.001  1000 | 0.047  0.136  1000 | 0.129  <0.001  1024 | 0.154  <0.001  1024 | 0.133  <0.001  1024 |
| **WBC** | *correlation*  *p-value*  *n* |  |  |  |  |  | 1 | 0.004  0.894  1138 | 0.260  <0.001  1138 | 0.068  0.021  1138 | 0.206  <0.001  1163 | 0.259  <0.001  1161 | 0.233  <0.001  1163 |
| **DEXA**  **bone** | *correlation*  *p-value*  *n* |  |  |  |  |  |  | 1 | 0.280  <0.001  1205 | 0.804  <0.001  1205 | 0.634  <0.001  1202 | 0.371  <0.001  1201 | 0.413  <0.001  1202 |
| **DEXA**  **fat** | *correlation*  *p-value*  *n* |  |  |  |  |  |  |  | 1 | 0.363  <0.001  1205 | 0.837  <0.001  1202 | 0.882  <0.001  1201 | 0.854  <0.001  1202 |
| **DEXA**  **lean** | *correlation*  *p-value*  *n* |  |  |  |  |  |  |  |  | 1 | 0.801  <0.001  1202 | 0.545  <0.001  1201 | 0.613  <0.001  1202 |
| **weight** | *correlaton*  *p-value*  *n* |  |  |  |  |  |  |  |  |  | 1 | 0.890  <0.001  1228 | 0.906  <0.001  1230 |
| **WC** | *correlation*  *p-value*  *n* |  |  |  |  |  |  |  |  |  |  | 1 | 0.903  <0.001  1228 |
