# Supplementary material for: Defining the end of puberty in boys: INSL3 and the acute determinants of adult Leydig-cell functional capacity
Source: Front Endocrinol (Lausanne). 2025 May 27;16:1574760. doi: 10.3389/fendo.2025.1574760 (PMC12148858; doi:10.3389/fendo.2025.1574760)
Supplement: Supplementary file 2 [file Table2.docx]

**Suppl. Table 2**

**Bivariate correlation analysis for inflammation and obesity-related parameters for young men aged 17 years.**

|  |  | **CRP** | **cotinine** | **DEXA**  **bone** | **DEXA**  **fat** | **DEXA**  **lean** | **weight** | **BMI** |
| --- | --- | --- | --- | --- | --- | --- | --- | --- |
| **INSL3** | *correlation*  *p-value*  *n* | -0.009  0.741  1242 | 0.039  0.279  770 | 0.032  0.266  1212 | -0.018  0.523  1212 | -0.041  0.157  1212 | -0.032  0.264  1227 | -0.059  0.038  1226 |
| **CRP** | *correlation*  *p-value*  *n* | 1 | 0.050  0.155  812 | 0.205  <0.001  1314 | 0.342  <0.001  1314 | -0.074  0.007  1314 | 0.303  <0.001  1331 | 0.344  <0.001  1329 |
| **cotinine** | *correlation*  *p-value*  *n* |  | 1 | -0.007  0.845  785 | -0.078  0.029  785 | -0.013  0.722  785 | -0.052  0.143  798 | -0.078  0.029  797 |
| **DEXA**  **bone** | *correlation*  *p-value*  *n* |  |  | 1 | 0.496  <0.001  1489 | 0.762  <0.001  1489 | 0.773  <0.001  1480 | 0.597  <0.001  1478 |
| **DEXA**  **fat** | *correlation*  *p-value*  *n* |  |  |  | 1 | 0.195  <0.001  1489 | 0.824  <0.001  1480 | 0.853  <0.001  14768 |
| **DEXA**  **lean** | *correlation*  *p-value*  *n* |  |  |  |  | 1 | 0.649  <0.001  1480 | 0.416  <0.001  1478 |
| **weight** | *correlaton*  *p-value*  *n* |  |  |  |  |  | 1 | 0.892  <0.001  1512 |
