# Supplementary material for: Defining the end of puberty in boys: INSL3 and the acute determinants of adult Leydig-cell functional capacity
Source: Front Endocrinol (Lausanne). 2025 May 27;16:1574760. doi: 10.3389/fendo.2025.1574760 (PMC12148858; doi:10.3389/fendo.2025.1574760)
Supplement: Supplementary file 3 [file Table3.docx]

**Suppl. Table 3**

**Relationship between smoking status by questionnaire and cotinine levels at 17 years.**

|  | cotinine concentration (ng/ml) | n | significance |
| --- | --- | --- | --- |
| regular smokers | 120.2 + 108.4 | 155 | p<0.001 |
| never smoked | 10.09 + 23.61 | 82 |  |
